# Supplementary material for: Impact of increased kidney function on clinical and biological outcomes in real-world patients treated with Direct Oral Anticoagulants
Source: PLoS One. 2022 Dec 9;17(12):e0278693. doi: 10.1371/journal.pone.0278693 (PMC9733869; doi:10.1371/journal.pone.0278693)
Supplement: Attachment — Submitted filename: Response to Reviewers.docx [file pone.0278693.s004.docx]

# Rebuttal letter

Dear Editors,

First of all, we would like to thank the referees and editors for the time they have invested in evaluating our paper, as well as for the valuable feedback they have provided.

Secondly, we would like to express our apologies if something was not clear in the original paper. We have now revised the document to better explain any possible misunderstanding.

The manuscript has been edited according to your concerns and all detailed responses to each comment are available hereunder.

We believe that the manuscript is now suitable for publication.

Melania Plaza Seijas,

On behalf of all the authors.

**Major Comments**

**1. The authors should notice the difference between (1) whether the superiority (or at least non-inferiority) of DOAC versus warfarin differs between patients with supranormal and normal kidney function levels and (2) whether efficacy of (either overall, or a specific type of) DOAC differs between patients with supranormal and normal kidney function levels. What the authors examined is actually the latter one, which is more relevant to investigate but obviously requires a larger sample size. The study is thus at risk of being underpowered. An analysis of statistical power is suggested as it is very likely that there could be difference of clinical outcomes between supranormal and normal kidney function levels, but the study failed to detect this simply due to the limited sample size. This limitation should also be discussed more in the discussion section, and the conclusion should be modified, because absence of evidence is not evidence of absence.**

*Answer: We performed the power analysis with the following results:*

*Power analysis for comparison of two incidence ratios are dependent of the observed incidence rates, and therefore slightly different for each event comparison (thrombosis event, hemorrhagic event, mortality, etc.). But approximately, with our sample size and follow-up time in each group, with an alpha error of 5% and a statistical power of 80%, the difference between the two given rates should be at least 5 events for every 100 patients/year in order to be able to detect it as statistically significant.*

*Also, it was added in the discussion section as follows:*

*The power of our data does not allow us to detect differences in complications of less than 5% patient-years. Consequently, although the difference in the incidences observed is null, we cannot guarantee with statistical certainty that they do not exist. But in this context, the no difference in the anticoagulant effect or plasma concentrations, especially at the pre-dose moment, allows us to postulate that if there is any clinical difference, it must be very small.*

**For example, the authors mentioned “Although edoxaban is not approved for patients with CrCl >95 mL/min in the USA [16], our data suggest that there are no significant differences in complication rates …”, but I don’t think the current study can serve as evidence against the FDA recommendation.**

*Answer: The sentence mentioned “Although edoxaban is not approved for patients…” is already changed: “Edoxaban is not approved for patients with CrCl >95 mL/min in the US due to its apparent worse effectiveness compared with warfarin in this group [17]. However, our data suggest that there is no increase in complication rates in patients with creatinine clearance higher than 90 mL/min neither in those treated with edoxaban or in those treated with DOACs.”*

**2. According to the inclusion/exclusion criteria presented in the method section, the study population is highly selected. This should be mentioned as a potential source of selection bias (i.e., limited generalizability). The study population mainly consisted of patients who were not “well” treated by VKA, while based on current international guidelines, DOAC is often the first recommended oral anticoagulant.**

*Answer: It is true that the population is highly selected, since a majority of the included patients were previously poorly controlled with VKA. However, we also think that it does not affect the topic or even the results that we are talking about in this paper.*

*This is mentioned in the discussion section:*

*“Our study population consisted mainly of patients who were not well treated by VKA since according to current international guidelines and the recommendations of the Spanish Health Authorities time in therapeutically range (TTR) must be higher than 65%. Opposite to many other countries, in Spain VKA is the first choice for patients with AF.* *TTR < 65 % is the most common reason for switching from VKA to DOACs. This could lead to a possible source of selection bias, although in our knowledge there is no relationship between renal function and INR variability.”*

**3. The study population were actually with GFR ≥30 mL/min, while in the manuscript it is mentioned several times that the investigated patients were with normal or supranormal kidney functions. Could a GFR <90 mL/min be termed as normal? This is misleading and should be corrected. It also raises a concern that it might be not appropriate to compare patients with GFR ≥90 mL/min to those with decreased GFR (given the research question the authors aimed to answer).**

*Answer: The reviewer is totally right about the confusion of talking about normal or supranormal kidney functions. We thought that maybe it is better to talk just about GFR <90 mL/min or >90 mL/min instead. We have corrected this in the entire manuscript.*

**4. More details should be added into the method section. The most important one is about the follow-up.**

*More details about the follow-up were added in the method section.*

**The study seems a cohort study, and I assume the authors evaluated the many inclusion/exclusion criteria at baseline. If so, would the patients be evaluated during the follow-up regarding the inclusion/exclusion criteria?**

*Answer: Yes, the inclusion/exclusion criteria was evaluated in patients at baseline and during the follow-up.*

**When would a patient be censored?**

*Answer: “Patients were followed up until DOAC treatment was discontinued, the patient died or decided to end his/her participation in the study.”*

**In real practice, patients may switch from DOAC to VKA, or stop receiving DOAC due to some medical reasons, and GFR may change as well. How did the authors handle these situations?**

*Answer: “**Patients whose renal function changed during follow-up were dose re-adjusted according to data sheet of each DOAC. Patients who switched from DOAC to VKA or simply stopped receiving DOAC* *and continued without anticoagulation ended the follow-up.”*

**The patients with GFR ≥90 mL/min had much shorter follow-up than those with GFR <90 mL/min, which is very likely to introduce bias (e.g., fewer clinical events were identified simply due to the shorter follow-up).**

*Answer: Although the median follow-up is slightly shorter in the patients with CrCl >90 mL/min (10 vs 13 months, Table1) this is already considered in the calculation of incidences, which uses total follow-up in each group (denominator into the incidence ratio, just obtained by the addition of all the individual follow-ups). In our opinion, by so doing, the possibility of bias in the estimation of incidences is really low.*

**In addition, more details should be introduced about how the studied clinical outcomes were determined (i.e., diagnosis criteria, recurrence, etc).**

*Answer: A new statement called: Outcomes measures was added. To better detail the diagnose of outcomes. See in Methods:*

*“Treatment outcomes were divided into effectiveness and safety. The primary effectiveness outcome comprised ischemic stroke and systemic embolism as major thrombotic complications and all-cause death during DOAC treatment, similar to the pivotal trials. The outcome clinically relevant non-major thrombosis (CRNMT) included myocardial infarction, transient ischemic attack, venous thromboembolism and superficial thrombophlebitis. Safety outcomes related to bleeding events were defined according to the Bleeding Academic Research Consortium (BARC) Scale: major hemorrhagic complications imply BARC type three to five and clinically relevant non-major bleeding (CRNMB) implies BARC type two. A composite outcome of major thromboembolic and major hemorrhagic complications was added to evaluate the overall risk of each DOAC. All outcomes were diagnosed in the hospital by standard imaging methods, i.e. cranial computerized, tomography (CT) scan, magnetic resonance imaging (MRI), Doppler ultrasonography, cardiac ultrasonography, and also by hemoglobin measurement and gastrointestinal or urinary endoscopy, in case of bleedings.”*

**5. The comparison of clinical outcomes between the two groups is at great risk of confounding. The statistical method the authors used is not helpful for this issue.**

*Answer: The reason of having used this statistical method (Fisher test) is because we wanted to keep the analysis as simple as possible. Performing multivariate analysis to adjust the data by other variables is risky when there are not enough events. Nevertheless, we performed a multivariate Cox survival analysis for the first event of thrombosis, hemorrhage, the combination of the two and death, adjusting by sex, age, CCI, BMI and CHA2DS2-VASc. In none of these four models the grouping variable “GFR ≥90 mL/min” was statistically significant (results not shown in the manuscript). In other words, the comparison between incidences is not confounded for any of these covariates.*

**Minor Comments**

**- Title: I suggest the authors consider to use a title which is less causal, such as “****Drug levels and clinical outcomes of patients treated with direct oral anticoagulants with supranormal kidney function”.**

*Answer: We thank the reviewer for this valuable suggestion and agree that it would be better to change it. “Drug levels and clinical outcomes of patients treated with direct oral anticoagulants with supranormal kidney function.”*

**- Keywords: Why did the authors only present edoxaban as a keyword but not the other three investigated DOACs?**

*Answer: The presented keywords were: kidney function; direct oral anticoagulants; glomerular filtration rate; edoxaban; thrombotic/hemorrhagic complications; anti-Xa activity. It is better to remove edoxaban as a keyword.*

**- Manuscript: About the presenting sequence of DOAC concentrations (or anti-Xa activity) and clinical outcomes, intuitively it is better to present DOAC concentrations first and then clinical outcomes, as the purpose of the abstract described “… focusing on biological parameters and thrombotic/hemorrhagic events”. At least, keep the sequence consistent throughout the manuscript.**

*Answer: Thank you for the comment, we totally agree with your opinion. The sequence has been changed in the abstract (results section) as it was easier to re-write it than doing so in the entire manuscript. So, we talk first about the DOAC concentrations/plasmatic effects (anti-Xa activity). And then about thrombotic/hemorrhagic events. The abstract now is as follows:*

*“Median follow-up [P25-P75] was 12.8 [7.6-21.2] months. 1,135 patients anticoagulated with DOACs were included and 152 patients with GFR >90 mL/min. Despite having significant differences in anti-Xa pre-dose (0.9 IU vs 0.12 IU/mL, p=0.006) and anti-IIa post-dose (160 ng/mL vs 300 ng/mL, p=0.050), which appear to be clinically irrelevant, no significant differences were observed in pre-dose (33,7 ng/mL vs 31,2 ng/mL, p=0.178) and post-dose (239,8 ng/mL vs 254,3 ng/mL, p=0.312) plasma concentrations of edoxaban.*

*Regarding the incidence of complications, of 18 serious thrombotic complications during follow-up, 17 (1.42 events/100 patient-years) occurred in patients with GFR <90 mL/min, and 1 (0.69 events/100 patient-years) in a patient with GFR >90 mL/min. A higher incidence of complications was observed in patients with <90 mL/min GFR, but the difference was not statistically significant (p>0.05). We found a total of 149 hemorrhagic complications. Forty-three were classified as severe bleeds (BARC grade 3 or more) and 38 (3.18 events/100 patient-years) of them occurred in the GFR <90 mL/min group, while the other 5 (3.43 events/100 patient-years) were in the GFR >90mL/min group. There were not statistically differences in the incidences between both groups (p=0.871).”*

**- Abstract: It is more informative to report the median follow-up and report it in the result section instead of the method section**.

*Answer: Thank you for the comment, it is true that it is better to report the median follow-up in the result section.*

*“Results: Median follow-up [P25-P75] was 12.8 [7.6-21.2] months.”*

**- Abstract: Outcome events that were investigated were not described (i.e., defined) in the method section. This makes it confusing in the result section that “A higher incidence of complications was observed…”**

*Answer: We totally forgot to talk about the outcome events, and we really appreciate your reminder to do so. We included a section in the material and methods section called “outcomes measures”.*

*“Outcome measures*

*Treatment outcomes were divided into effectiveness and safety. The primary effectiveness outcome comprised ischemic stroke and systemic embolism as major thrombotic complications and all-cause death during DOAC treatment, similar to the pivotal trials. The outcome clinically relevant non-major thrombosis (CRNMT) included myocardial infarction, transient ischemic attack, venous thromboembolism and superficial thrombophlebitis. Safety outcomes related to bleeding events were defined according to the Bleeding Academic Research Consortium (BARC) Scale: major hemorrhagic complications imply BARC type three to five and clinically relevant non-major bleeding (CRNMB) implies BARC type two. A composite outcome of major thromboembolic and major hemorrhagic complications was added to evaluate the overall risk of each DOAC. All outcomes were diagnosed in the hospital by standard imaging methods, i.e. cranial computerized, tomography (CT) scan, magnetic resonance imaging (MRI), Doppler ultrasonography, cardiac ultrasonography, and also by hemoglobin measurement and gastrointestinal or urinary endoscopy, in case of bleedings.”*

**In addition, hemorrhagic complications were only mentioned in the conclusion.**

*Answer: Thank you for the comment, it is true that hemorrhagic complications were only mentioned in the conclusion. Now, we talked about hemorrhagic complications in the results as well as in the conclusion. In the results section there is the following statement:*

*“We found a total of 149 hemorrhagic complications. Forty-three were classified as severe bleeds (BARC grade 3 or more) and 38 (3.18 events/100 patient-years) of them occurred in the GFR <90 mL/min group, while the other 5 (3.43 events/100 patient-years) were in the GFR >90mL/min group. There were not statistically differences in the incidences between both groups (p=0.871).”*

**- Abstract: The “normal” range of GFR should be presented, instead of “<90mL/min” only**.

*Answer: The “normal” range of GFR was added in methods section of the abstract. “**In our laboratory, normal range of GFR is 60-90 mL/min.”*

**- Abstract: The percentages of serious thrombotic complications should also be presented for both groups, instead of the absolute numbers only.**

*Answer: We agree that percentages of serious thrombotic complications as well as the absolute numbers can give a more accurate information. You can see the following statement in the abstract:*

*“Regarding the incidence of complications, of 18 serious thrombotic complications during follow-up, 17 (1.42 events/100 patient-years) occurred in patients with GFR <90 mL/min, and 1 (0.69 events/100 patient-years) in patients with GFR ≥90 mL/min.*

**- Abstract: It is still informative to present some numbers of the results of DOAC concentrations (or anti-Xa activity) between groups in the abstract, instead of simply mentioning “No statistically significant differences”, as the study is at risk of being underpowered.**

*Answer: We agree that it is more informative to present the numbers and maybe the p-value of DOAC concentrations and anti-Xa activity. They are now added in order to clarify the non-statistically significant differences:*

*“Despite having significant differences in anti-Xa pre-dose (0.09 IU vs 0.12 IU/mL, p=0.006) and anti-IIa post-dose (160 ng/mL vs 300 ng/mL, p=0.050), which appear to be clinically irrelevant, no significant differences were observed in pre-dose (33,7 ng/mL vs 31,2 ng/mL, p=0.178) and post-dose (239,8 ng/mL vs 254,3 ng/mL, p=0.312) plasma concentrations of edoxaban.”*

**- Abstract: Please rephrase the conclusion in the abstract. Some details are better to present in the result section, such as “including 66% treated with edoxaban”.**

*Answer: The conclusion in the abstract has been rephrased:*

*“In our patients treated with DOACs and with a GFR >90 mL/min we did not observe an increased incidence of thrombotic/hemorrhagic complications. Likewise, drug anti-Xa activity and edoxaban plasma concentration did not seem to be influenced by GFR.”*

**- Introduction: How other types of DOACs are eliminated should also be introduced.**

*Answer: It is true that only edoxaban elimination was reported. We added the elimination of all the DOACs (apixaban, dabigatran, edoxaban and rivaroxaban):*

*“Apixaban is 73% excreted by the liver and 27% excreted by the kidney. Dabigatran is 20% and 80% excreted by the liver and the kidney respectively. Edoxaban is 50% excreted by the liver and 50% by the kidney. Finally, rivaroxaban is 65% excreted by the liver and 35% by the kidney.”*

**- Introduction: The reference 5 was not the ENGAGE AF-TIMI 48 trial.**

*Answer: Our apologies for the mistake with the reference. We made the correction:*

*“[5] Giugliano RP, Ruff CT, Braunwald E, Murphy SA, Wiviott SD, Halperin JL, et al. ENGAGE AF-TIMI 48 Investigators (2013). Edoxaban versus warfarin in patients with atrial fibrillation. N Engl J Med;369:2093-2104.”*

**“The ENGAGE AF-TIMI 48 study [5], …, reported a higher ischemic stroke rate for edoxaban in patients with CrCl >95 mL/min (hazard ratio (HR) [95% confidence interval (CI)]: 1.45 (0.90-2.35); p=0.13).” This sentence and the HR here is difficult to understand without reading the manuscript that reported the trial, as the trial compared Edoxaban with Warfarin, while the interested comparison for the current manuscript is Edoxaban in patients with supranormal GFR versus normal GFR who are both treated with Edoxaban (see my first comment). For the same reason, the paragraph 2, 5, and 6 of the introduction are not that relevant to the current study.**

*Answer: In our opinion, both things are helpful to introduce the reader into the main subject, which is to compare, in real-life, biological parameters and thrombotic/hemorrhagic complication rates in DOAC-treated patients with GFR >90 mL/min vs GFR <90mL/min.*

**In addition, I suggest to introduce more evidence based on which FDA and EMA made the recommendations, and explain why the current study is still relevant to conduct given the existing recommendations.**

*Answer: It is true that more evidence would explain better the relevance of the current study. In the introduction, this evidence was added based on FDA and EMA recommendations:*

*“As mentioned before, approximately half of the edoxaban dose is eliminated by the kidney, and edoxaban blood levels are lower in patients with better renal function, averaging about 30% less in patients with CrCL of > 80 mL/min, and 40% less in patients with CrCL > 95 mL/min. The FDA mentioned that “as renal function improves and edoxaban blood levels decrease, the risk for ischemic stroke increases in patients with NVAF [13]. In addition, the European Medicines Agency (EMA) issued the recommendation that “edoxaban should be used only in patients with high CrCl after careful evaluation of individual thromboembolic and bleeding risk” [14].”*

**- Introduction: “DOACs for patients with an increased GFR (CrCl >95 mL/min) have been reported to be less effective, as either the desired therapeutic range is not reached or the drug is more rapidly eliminated.” Citations of these reports are necessary.**

*Answer: We have realized that the sentence was not correct, so we made the following correction both for the statement and for the citation:*

*“DOACs for patients with an increased GFR (CrCl >95 mL/min) have been reported to be less effective. Nevertheless, this conclusion comes from a nationwide study where patients taking Edoxaban 30mg and having a CrCl >95 mL/min presented higher incidence of stroke than warfarin treated patients [10]. This suggests that either the desired therapeutic range is not reached or the drug is more rapidly eliminated.”*

*“[10] Hee Tae Yu, MD, PhD*; Pil-Sung Yang, MD*; Tae-Hoon Kim, MD; Eunsun Jang, MS; Daehoon Kim, MD; Jae-Sun Uhm, et al. Impact of Renal Function on Outcomes With Edoxaban in Real-World Patients With Atrial Fibrillation. A Nationwide Cohort Study. Stroke 2018;2421-2429. DOI: 10.1161/STROKEAHA.118.021387.”*

**- Method: Please add the ethic approval number for the study. If it is the same as that of the project number, this should be mentioned in the method section.**

*Answer: The ethic approval number is now included: “(IIBSP-ACO-2018-21)”.*

**- Method: It is incorrect that the CHA2DS2-VASc score is used for evaluating risk of VTE (instead, for ischemic stroke in AF patients). The below text in the manuscript should be corrected: “… treated with DOACs to prevent stroke or systemic embolism due to their high VTE risk (CHA2DS2-VASc score ≥2)”;**

*Answer: We agree with the comment, so we changed each sentence as you can see below:*

*“Included were patients aged >18 years, with AF treated with DOACs to prevent stroke (CHA2DS2-VASc score ≥2) [24] or with recurrent venous thromboembolism (VTE).”*

**“… VTE risk for patients with AF (CHA2DS2-VASc) …”;**

*Answer:* ***“…****ischemic stroke risk in AF patients (CHA_2_DS_2_-VASc score).”*

**“… and had a CHA2DS2-VASc score on average one point higher, indicating a higher VTE risk.”**

*Answer: “…and had a CHA2DS2-VASc score on average one point higher indicating a higher thrombotic risk.”*

**- Discussion: I don’t think it’s correct that “(limited sample size and short follow-up) is also strength, as this made personalized follow-up of patients possible and so was a good guarantee of complications being reported”.**

*Answer: Thank you for your comment. We rewrite the sentence and now it might be clearer and more understandable as we keep thinking that it can also be a strength in our study.*

*“However, the fact of having a small sample size could also be considered a strength (in comparison with giant real-world registries) because all the patients were followed-up by the same clinical team, the same protocol was implemented, and the complications record was very exhaustive, ensuring an accurate estimation of the incidence of complications.”*

**- Manuscript: There are some grammar errors, or sentences that seem not natural to the English language. I list some of them below, but please carefully read the full text and try to improve the language.
--- Abstract: “… a higher thromboembolic events incidence” -> ““… a higher incidence of thromboembolic events”;
--- Abstract: “Observational prospective single-centre study and registry of patients on DOACs.” This is not a sentence;
--- Abstract: “Anti-Xa activity … was measured for edoxaban, apixaban, and rivaroxaban; diluted thrombin time for dabigatran …; and additionally, plasma concentrations in edoxaban …” Please rephrase this sentence;
--- Introduction: “Since direct oral anticoagulant (DOAC) drugs” -> remove the “drugs”;
--- Method: “… and included patients signed an informed consent” -> “… and included patients who signed an informed consent”.**

*Answer: All the grammar errors were corrected. Thank you very much for your insightful suggestions and comments!*
